# The efficacy of 3D gait analysis to evaluate surgical (and rehabilitation) outcome after degenerative lumbar surgery

**DOI:** 10.1186/s12893-024-02486-0

**Published:** 2024-06-26

**Authors:** Chao Zhou, Ning Zhou, Yanping Zheng, Haipeng Si, Yanguo Wang, Jun Yin

**Affiliations:** 1 Department of Spine Surgery, Cheeloo College of Medicine, Qingdao Medical Engineering Interdisciplinary Key Laboratory, Qilu Hospital (Qingdao), Shandong University, No.758, Heifei Road, Qingdao, Shandong 266000 China; 2 Intensive Care Unit, Binzhou Central Hospital, No. 108, Huancheng South Road, Huimin, Binzhou, Shandong 251700 China

**Keywords:** Lumbar degenerative disease, Three-dimensional gait, Gait analysis, Rehabilitation, Correlation

## Abstract

**Background:**

Lumbar degenerative conditions are a major cause of back pain and disability in individuals aged 45 and above. Gait analysis utilizes sensor technology to collect movement data, aiding in the evaluation of various gait aspects like spatiotemporal parameters, joint angles, neuromuscular activity, and joint forces. It is widely used in conditions such as cerebral palsy and knee osteoarthritis. This research aims to assess the effectiveness of 3D gait analysis in evaluating surgical outcomes and postoperative rehabilitation for lumbar degenerative disorders.

**Methods:**

A prospective self-controlled before-after study (*n* = 85) carried out at our Hospital (Sep 2018 - Dec 2021) utilized a 3D motion analysis system to analyze gait in patients with lumbar degenerative diseases. The study focused on the multifidus muscle, a crucial spinal muscle, during a minimally invasive lumbar interbody fusion surgery conducted by Shandong Weigao Pharmaceutical Co., Ltd. Pre- and postoperative assessments included time-distance parameters (gait speed, stride frequency, stride length, stance phase), hip flexion angle, and stride angle. Changes in 3D gait parameters post-surgery and during rehabilitation were examined. Pearson correlation coefficient was employed to assess relationships with the visual analog pain scale (VAS), Oswestry Disability Index (ODI), and Japanese Orthopedic Association (JOA) scores. Patient sagittal alignment was evaluated using “Surgimap” software from two types of lateral radiographs to obtain parameters like pelvic incidence (PI), pelvic tilt (PT), sacral slope (SS), lumbar lordosis (LL), intervertebral space height (DH), posterior height of the intervertebral space (PDH) at the operative segment, and anterior height of the intervertebral space (ADH).

**Results:**

By the 6th week post-operation, significant improvements were observed in the VAS score, JOA score, and ODI score of the patients compared to preoperative values (*P* < 0.05), along with notable enhancements in 3D gait quantification parameters (*P* < 0.05). Pearson correlation analysis revealed a significant positive correlation between improvements in 3D gait quantification parameters and VAS score, JOA score, and ODI value (all *P* < 0.001).

**Conclusion:**

3D gait analysis is a valuable tool for evaluating the efficacy of surgery and rehabilitation training in patients.

## Introduction

Lumbar degenerative diseases are the leading cause of low back pain and the first leading cause of disability at age above 45 years and the second most common reason for primary healthcare visits [1, 2]. The diseases might be presented as disc herniation, facet joint arthropathy, lumbar spinal stenosis, or combinations of them. Imaging characteristics of degenerative disc disease include narrowed disc space, weak or complete loss of T2 signal intensity in the disc space, ligamentous alterations, altered bone marrow, herniation, osteophyte formation, stenosis, and subluxation [3–6]. Conservative therapy is primarily utilized to treat patients with early IDD in order to alleviate lower back pain and improve their quality of life. However, it is important to note that conservative therapy is considered palliative and cannot cure the condition [7]. On the other hand, there are various medications commonly used to treat LBP, including NSAIDs, opioid analgesics, muscle relaxants, benzodiazepines, antidepressants, corticosteroids, and antiepileptic drugs [8]. In addition to medication, non-pharmacological interventions such as bed rest, traction, bracing, exercise therapy, acupuncture, massage, electromagnetic or thermal therapy, and psychotherapy can also be utilized. These methods are often combined with medication or surgery across various disciplines [9]. Advancements in endoscopic technology have improved precision in direct visual operations, making intervertebral disc fusion a standard surgical option for symptomatic IDD. Both minimally invasive and open surgeries offer benefits like reduced muscle edema and improved postoperative recovery [10]. Overall, the treatment approach for IDD depends on the severity of the condition, the effectiveness of conservative therapy, and the individual patient’s needs and preferences [11, 12].

In recent years, various minimally invasive techniques have been developed for the treatment of lumbar degenerative diseases [13–18]. Posterior lumbar interbody fusion (PLIF) and transforaminal lumbar interbody fusion (TLIF), two main techniques in lumbar interbody fusion, have been developed to provide solid fixation of spinal segments [19–27]. Both of them yield good outcomes in the treatment of degenerative spondylolisthesis. Minimally invasive techniques in TLIF surgery reduce the occurrence of iatrogenic lumbar back muscle injury [26, 28–30]. Different research groups have reported that minimally invasive transforaminal lumbar interbody fusion has high feasibility and safety in the treatment of lumbar degenerative diseases [14, 16, 17, 21, 31–34].

The clinical assessment of the treatment outcomes in spinal diseases includes VAS [35], ODI [36], standing-walk timing test to assess muscle strength, gait, and balance ability in community-dwelling older individuals [37], and Berg balance scale [38]. The VAS score can effectively reflect the intensity of pain, the ODI and Berg Balance scale can evaluate the subject’s dysfunction and balance function, respectively, and the stand-up-walk timing test can reflect the subject’s ability to balance, walk and other functional movements [39]. Although these evaluation indicators have a wide range of applications, they suffer different drawbacks including the subjectivity of the evaluation results, which cannot guarantee the accuracy, specificity, and objectivity of the evaluation outcomes. Therefore, an objective and quantifiable measurement method still needs to be explored clinically [40].

Advanced measurement techniques can establish kinematic variables, but basic spatiotemporal gait characteristics can be estimated with minimal equipment. On the other hand, Wearable technology is affordable and portable, making it a popular fitness trend globally. It benefits not only the healthy but also the elderly and those with chronic illnesses. Wearable technology allows for real-world measurement of people’s movements across different demographics. Recent systematic reviews have highlighted this trend, with sensors like accelerometers, gyroscopes, and magnetometers commonly used as inertial measurement units to quantify gait patterns, providing a cost-effective alternative to lab-based methods [41]. Gait analysis is a technique to study human gait movement function by using various sensor technologies to obtain kinematics and electromyographic signals of human lower limbs, and to analyze their time and space characteristics [41–44]. Gait analysis can provide a wide range of gait characteristics, including spatiotemporal parameters, dynamic joint range of motion angles, neuromuscular activity, dynamic joint reaction forces, etc [38, 45]. These parameters can be evaluated in two-dimensional (2D) or three-dimensional (3D) planes to evaluate the characteristics of specific anatomical planes [46, 47]. At present, gait analysis is widely used in the fields of cerebral palsy [48], and knee osteoarthritis [49]. Functional scales including VAS score and ODI are commonly used worldwide to evaluate the effectiveness of treatments for spinal diseases. However, it is frequently challenging to be precise and thorough due to the subjectivity of these assessment techniques. Thus, research in the real world on therapeutically applicable, objective, and quantitative assessment techniques is still required. The study investigates the use of 3D gait quantitative analysis in evaluating surgical efficacy and postoperative rehabilitation in lumbar degenerative disease patients. It examines changes in gait parameters, kinematics, imaging, functional scales, and multifidus cross-sectional area before and after surgery.

## Patients and methods

### Patients

This was a prospective self-controlled before-after study conducted on patients with lumbar degenerative disease (*n* = 85) admitted to our Hospital between September 2018 and December 2021. The patients (*n* = 85) with lumbar degenerative diseases including 42 males and 43 females; aged 18–84 years, aged (53.32 ± 18.12) years old; Body mass index (BMI) 18.7–26.9 kg/m^2^, with an average value of 23.36 (SD ± 1.84) kg/m2; disease types: In this study, we had 15 cases of spondylolisthesis, 36 cases of lumbar spinal stenosis, 30 cases of lumbar disc herniation, and 4 other patients with clinical symptoms of lumbar degeneration, such as lumbar and leg pain, lumbar discomfort according to the physical examination, clinical symptoms and paraclinical findings.

Inclusion criteria of the study were age ≥ 18 years old, presence of lumbar spondylolisthesis, spinal stenosis or lumbar intervertebral disc and other diseases confirmed by positive computed tomography (CT) or magnetic resonance imaging (MRI), meeting the surgical indications including minimally invasive transforaminal lumbar interbody fusion, course of disease > 3 months, no significant improvement in conservative treatment such as management therapy, traction, and massage, the straight leg raising test is all (+); there are clinical symptoms of lumbar degenerative diseases such as intermittent claudication, lumbar discomfort, lumbago and leg pain, and muscle loss symptoms, signed informed consent voluntarily. The exclusion criteria were BMI > 27.5 kg/m ^2^ (obesity) [50], presence of visual impairment or vestibular function-related diseases that affect walking and balance, lumbar spinal canal tumors, congenital lumbar deformities, previously received lumbar surgery, having old or fresh spinal fractures, previous medical history of ankylosing spondylitis, congenital lumbar scoliosis, thoracic kyphosis, lumbar spine-specific infection, severe osteoporosis, lumbar tuberculosis, lumbar fracture, multi-segment fusion required, with the vertebral body, spinal canal, intervertebral space and paraspinal infection; lumbar scoliosis > 10 °, neurogenic disease or myogenic disease, coagulation dysfunction, severe cardiovascular and cerebrovascular diseases, sequelae of cerebrovascular diseases or meniscus injury, knee arthritis or other lower limb diseases affecting walking posture, pelvic and hip joint deformities, and lost to follow-up after surgery.

### Method

All experimental procedures of this study were approved by the local ethics committee, which were in complete accordance with the regulations and ethical standards of the study on human beings set by the Helsinki Declaration (2014) [51]. All procedures of the study, experiments and possible risks and benefits from the experiments were clearly explained to the patients. All patients signed a written informed consent form for participating in the study. Informed consent was obtained from medical authorities for using the medical records and laboratory tests of the subjects in this study.

### Surgical procedure

**Fig. 1:**
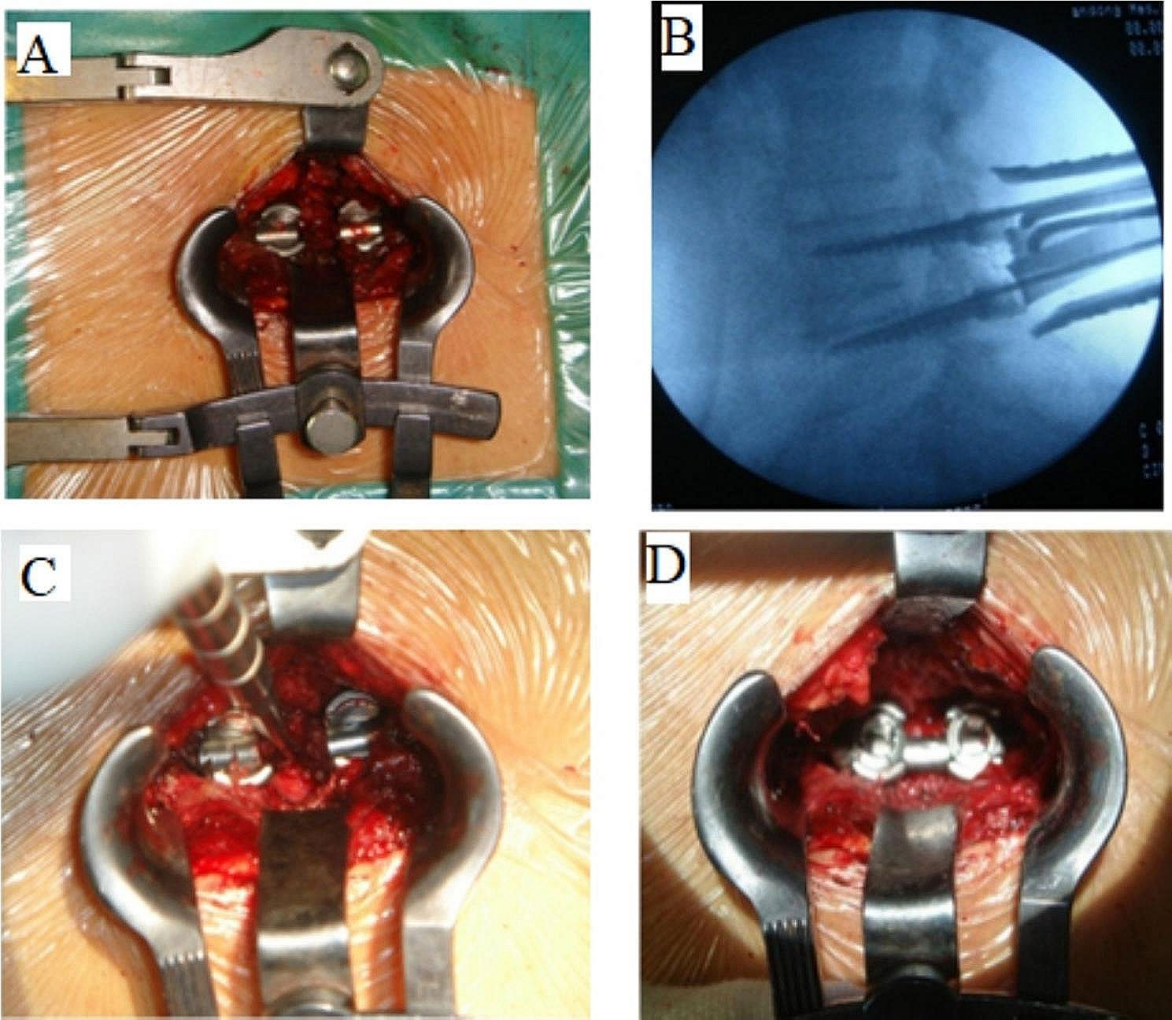
The Operative procedures of the surgical operation via the Wiltse approach. (**A**) Incision of thoracolumbar fascia is made from the side of the multifidus and longissimus.; (**B**) Placement of retractor between multifidus and longissimus muscle.; (**C**) Facet joints and laminae are removed to reveal the intervertebral disc.; (**D**) Fixation of the rod and screws

**Fig. 2:**
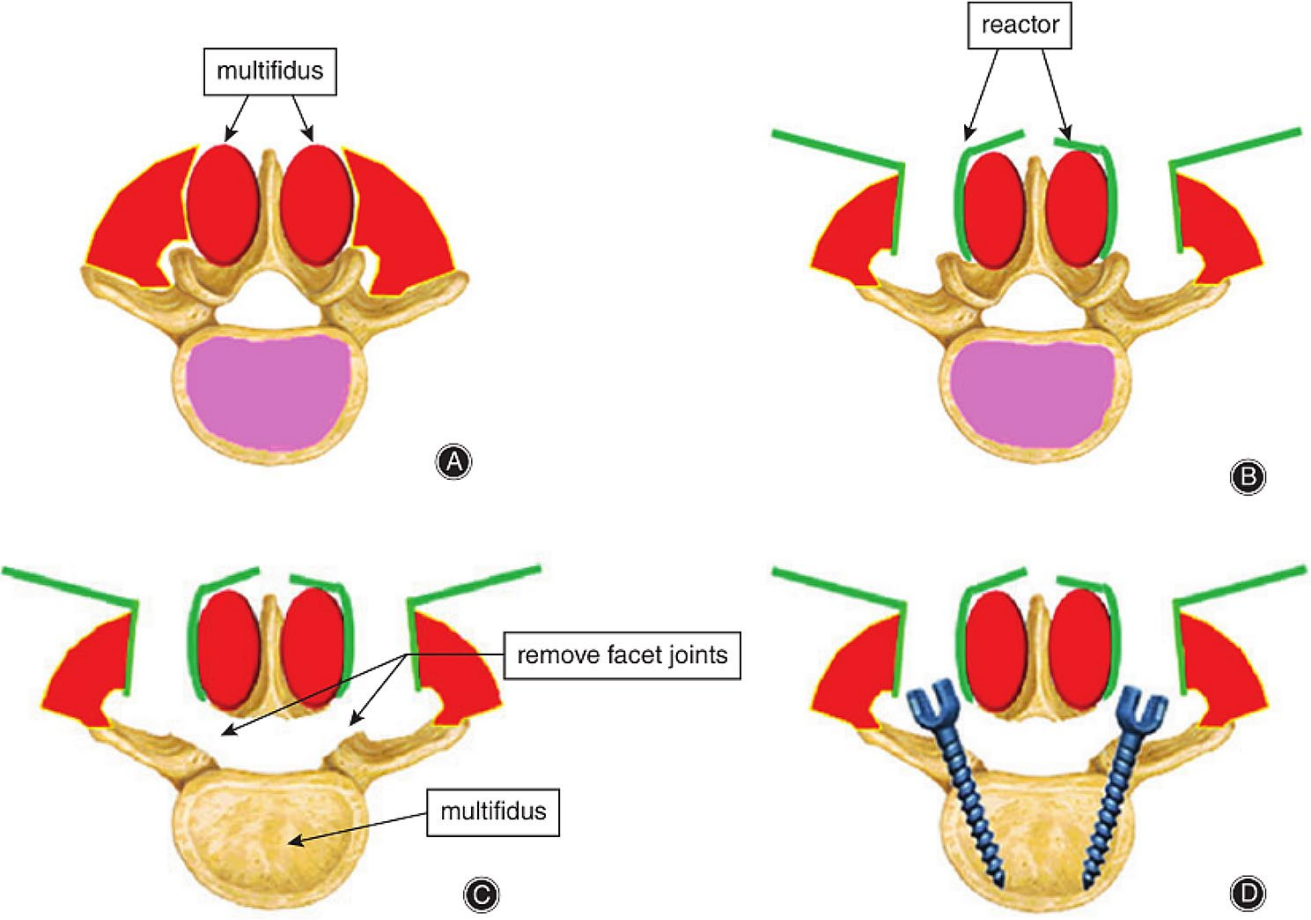
Schematic diagram of the surgical operation through the Wiltse approach. (**A**) Incision of thoracolumbar fascia is made from the side of the multifidus and longissimus. (**B**) Placement of retractor between multifidus and longissimus muscle. (**C**) Facet joints and laminae are removed to reveal the intervertebral disc. (**D**) Fixation of the rod and screws

Shandong Weigao Pharmaceutical Co., Ltd. Co. performed a minimally invasive lumbar interbody fusion through the lateral space of the multifidus muscle using the Wiltse approach. The procedure involved general anesthesia, a prone position, and a sterile drape. The lesion segment of the lumbar spine and surface projection points of the upper and lower pedicles were determined under fluoroscopy. A longitudinal incision was made, and the skin, subcutaneous tissue, and nerve fascia were cut layer by layer. A positioning guide wire was inserted through the intermuscular space of the multifidus muscle, and the muscle fibers were bluntly separated. A pipeline dilator was inserted along the expansion cannula, and the operative field was cleaned. The intervertebral fusion cage was filled with autogenous bone, and the pedicle screw was placed using a UPASS II nail bar. Figures 1 and 2 illustrate the surgery procedure.

### Rehabilitation training

Based on the features of PELD recovery, the rehabilitation program was split into three phases and carried out between weeks 1 to 2, 2 to 6, and 6 to twenty-four. The major goals of this program were to prevent complications from surgery by using lower extremity exercise, to reduce intervertebral disc stress in the early stages through active spine extension, to increase muscle flexibility in the middle stages through muscle stretching, to strengthen core muscles and correct posture and gait in the later stages. Specific operations were as follows: (a) Bed rest and passive activities such as heat or cold therapy and passive stretching with minimal stretches of the muscles and soft tissues around the lumbar spine to improve flexibility should be given priority within 1 week after surgery (b) Active activities in bed should be implemented from the second week for the lower extremities in bed, straight leg raising training and isometric contraction training for lumbar extensors. A waist girdle or lumbosacral brace was used to stand by the bed for 10 min 2–3 times a day. (c) -6 weeks to carry out active stretching exercises for the spine, gradual stretching exercises for the lower back muscles, etc., to promote symptom relief and increase joint flexibility or mobility and muscle elasticity; (d) After 6 weeks, intensive training was performed to increase core muscle strength, and training in the walking force mode. The intense training program included (a) training for waist and hip muscles, such as bridge exercise, supine arching exercise, and sitting forward bending; (b) training for abdominal muscles, such as lying upper limb support exercise, supine leg raising exercise and improved Yanfei exercise; (c) Training for pelvic and lower limb functions, such as lateral pelvic strengthening exercises in the supine position, alternate pelvic pronation exercises in the supine position and crawling training.

### Three-dimensional gait quantitative analysis

**Fig. 3:**
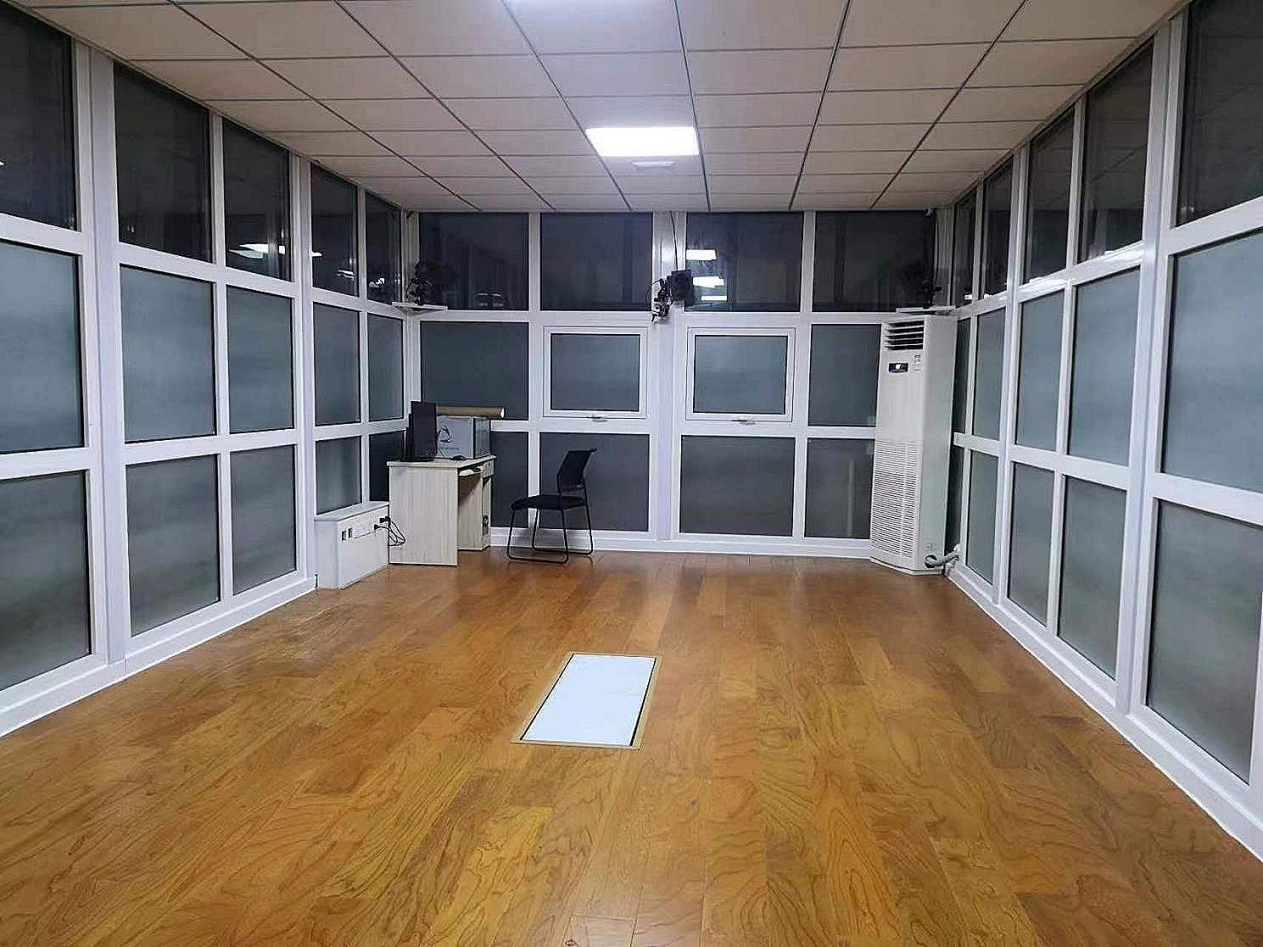
Overview of the three-dimensional gait analysis laboratory

**Fig. 4:**
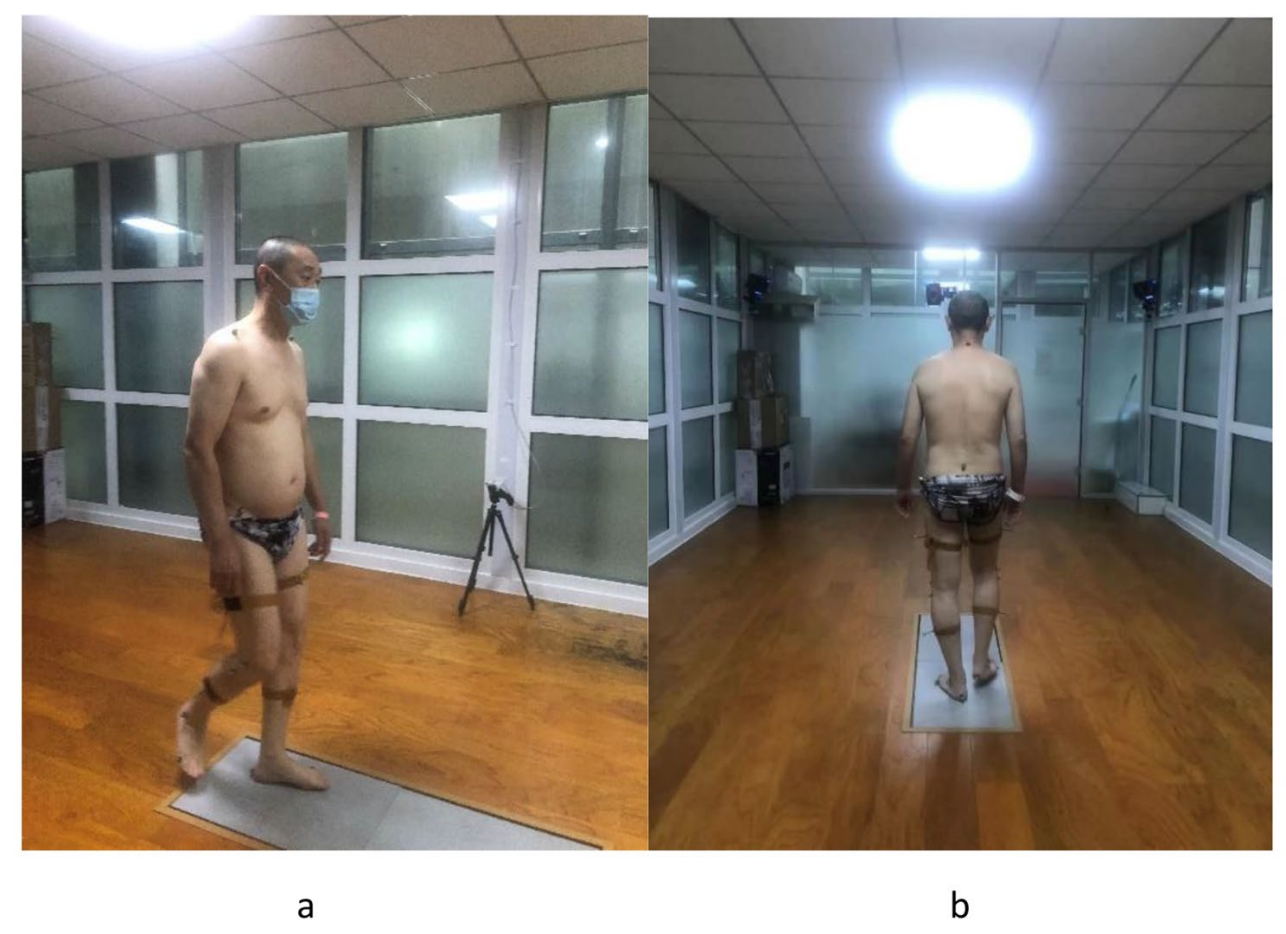
Placement of 22 fluorescence tracking points according to the requirements of the DAVIS HEEL template. The patients underwent the 3D gait quantitative analysis system. According to the fluorescent marker placement standard of the DAVIS HEEL template, a fluorescent marker point was pasted on the subject. Fluorescent markers fixed on the calf and thigh with elastic bandages were placed on the outside of the patient’s lower limbs as tracking points, with a total of 22 points. Figs a and b illustrate the patient’s position during the test

**Fig. 5:**
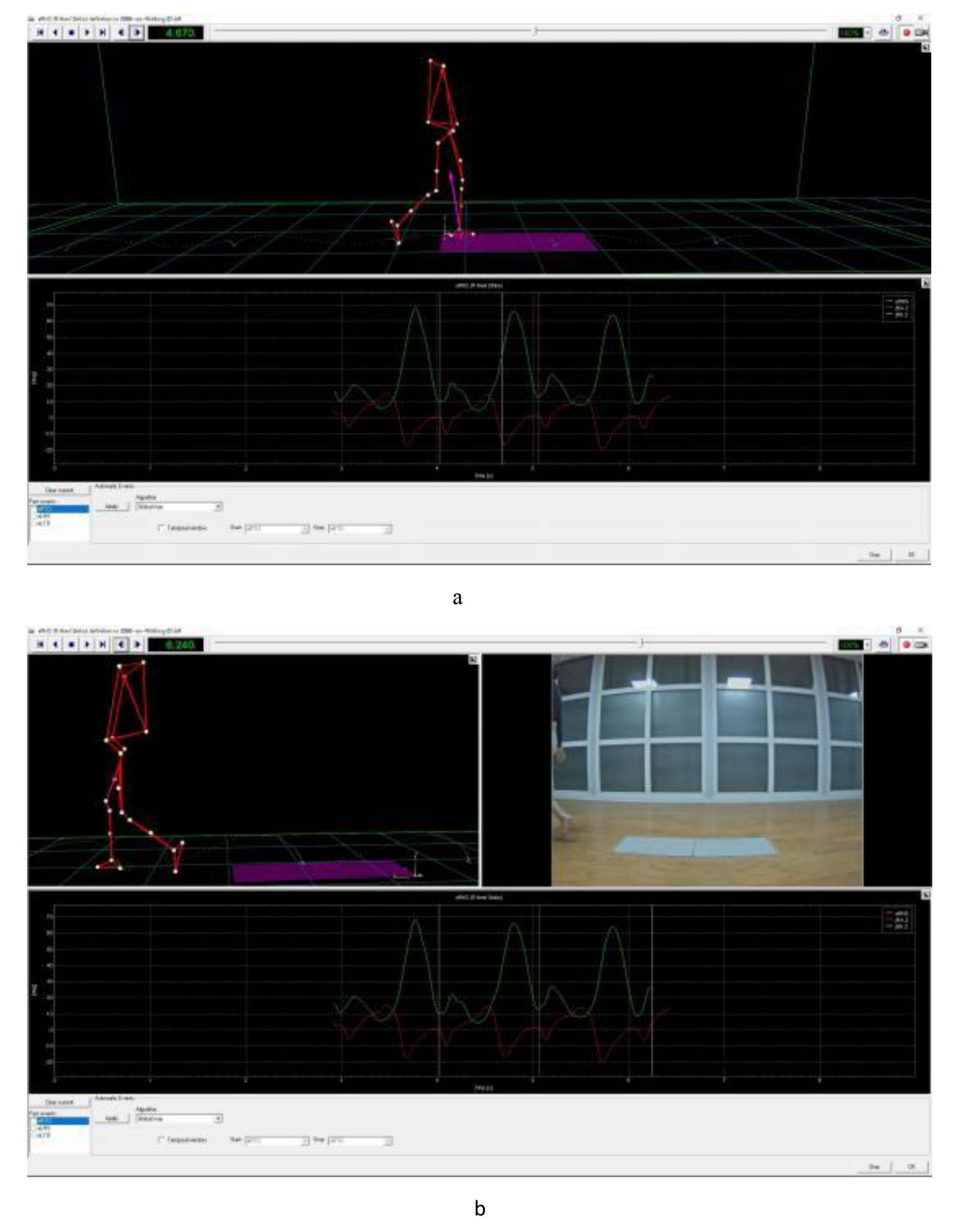
Division process of the gait cycle. Normal gait consists of two phases: the stance phase; and the swing phase. These phases are further divided into various sub-phases. Also, the gait cycle involves a combination of open- and closed-chain activities [43]. **a** and **b** illustrate these cycles

The patients underwent a 3D gait quantitative analysis system examination before the operation and 6 months after the operation. According to the fluorescent marker placement standard of the DAVIS HEEL template, a fluorescent marker point with a diameter of 14 mm was pasted on the subject. The position placement used the markers as follows: (a) Trunk markers: left and right acromion points, spinous process of the seventh cervical vertebra; (b) Pelvic landmarks: left and right anterior superior iliac spines, upper border of the second sacral vertebra; (c) lower extremity landmarks: left and right greater trochanters, medial and medial epicondyles of the femur, middle of the calf, middle of thigh, inner and outer malleolus, dorsal border of head of fifth toe, heel point. Fluorescent markers fixed on the calf and thigh with elastic bandages were placed on the outside of the patient’s lower limbs as tracking points, with a total of 22 points. During the test, all subjects wore light tops and tight shorts, and took off their shoes. Before the test, the professional technicians will explain the test process, test principle and test precautions to all the subjects with unified instructions, and give the subjects sufficient time to adapt to the environment and familiarize themselves with the aisle. The subjects were asked to perform a series of 5 ground walking tests at a normal and natural speed on a 30 m long flat ground, and the whole gait cycle was taken from the middle of the 5th test for kinematic analysis. There is only one foot data on each force plate and the foot is completely stepped on a force plate as valid data. Note: During the process of collecting gait data, if the fluorescent marker falls off, re-collect the gait data. The collected gait data are named fluorescent marker points in the system, and the gait cycle of each collected gait is carefully divided according to the time phase of two heel strikes and toe off the ground, and then the gait collection values are consistent for five times. Sexual analysis to remove the collected data with large deviation. Then, generate this gait detection report and export the report sheet [52]. Figure 3 illustrates an overview of the three-dimensional gait analysis laboratory used in this study. Figure 4 shows the placement of 22 fluorescence tracking points according to the requirements of the DAVIS HEEL template. The division process of the gait cycle is presented in Fig. 5.

### Pelvis-lumbar spine imaging parameters

All patients underwent standing full-length lateral spine and lateral lumbar spine X-ray radiographs preoperatively and 6-month post-operatively in a standardized position [53] to evaluate the preoperative and postoperative sagittal alignment parameters and Roussouly classification. An independent experienced spine surgeon reviewed the clinical data, which included general data (gender, age, and follow-up time); the patient’s diagnosis; the number of surgery segments needed; and the preoperative and postoperative balance parameters of lumbosacral dislocation, including pelvic incidence (PI), pelvic tilt (PT), sacral slope (SS), lumbar lordosis (LL), intervertebral space height (DH), the posterior height of the intervertebral space (PDH) at the operative segment, and the anterior height of the intervertebral space (ADH) at the operative segment.

Two experienced spinal surgeons assessed and measured the radiographic parameters via Surgimap version 2.2.13.1 software (The Physician Driven Image Solution, Nemaris Inc., New York, NY, USA) on Windows as per the previously described methods [24, 54]. The interobserver agreement (Kappa) was calculated according to the following formula: Kappa= (Pr(a)- Pr(e))/ (1-Pr(e)), where Pr(a) represents the actual observed agreement, and Pr(e) represents chance agreement. The interobserver agreement according to the Kappa value was equal to 0.91 [55].

### Routine rehabilitation assessment

#### Pain assessment

The Visual Analogue Scale (VAS) was used to evaluate the pain improvement of patients before the operation and 6 months after the operation. VAS score was used to assess the improvement of the patient’s pain, which was graded from mild to severe (0–10 points) based on the patient’s subjective pain degree. The higher the score, the more intense the pain [43].

#### Evaluation of low back pain

The Japanese Orthopedic Association (JOA) score was used to evaluate the low back pain of patients before the operation and 6 months after the operation, including subjective symptoms (lower back pain, leg pain and/or tingling pain, gait), and clinical parameters [56]. There are 4 dimensions of physical signs (straight leg raising test, sensory impairment, and motor impairment), limitation of daily activities, and bladder function. The highest score for subjective symptoms is 9 points, the highest score for daily activities is 14 points, and the highest score for bladder function is 6 points. The total score is 29 points. The lower the score, the more severe the dysfunction of the patient [43].

#### Degree of dysfunction

The ODI scale was used to measure the degree of dysfunction of patients before the operation and 6 months after the operation. ODI score was used to evaluate pain, single function and individual comprehensive function. There are 9 items, and each item is scored from 0 to 5, with a maximum score of 45. The higher the score, the more severe the functional impairment [36].

#### Cross-sectional area of multifidus

The 1.5 T scanner (Achieva, Philips Medical Systems, Best, and The Netherlands) was used for all lumbar and cervical spine MRI scans. The T2-weighted scans of the axial intervertebral space level from the MRI image of the lumbar spine operation segment were obtained before the operation and 6 months after the operation. The cross-sectional boundary of the multifidus muscle was selected by freehand tracing, and its area was calculated as per the methods described in previous studies [57, 58].

### Observation indicators

Observation of gait time-space parameters, kinematics-joint range of motion and pelvic-lumbar imaging parameters, VAS, JOA and ODI scores, multifidus cross-section of 85 patients before operation and 6 months after operation. The correlations of 3D gait quantitative parameters with pelvic-lumbar imaging parameters, VAS, JOA and ODI scores, and multifidus cross-sectional area were evaluated by Pearson correlation coefficient.

### Statistical analysis

Statistical analyses of the study were conducted with Statistical Package for Social Sciences (IBM SPSS Statistics Inc., Chicago IL, Windows version 23.0). At first, the normality of all the continuous variables was assessed with the Kolmogorov-Smirnov normality test. The variables with normal distribution were presented as means ± standard deviation ($\bar x$± *s*) and variables with skewed distribution were presented in median (Inter Quartile Range, IQR). For normal distributed variables, the comparison between groups was performed by One-way ANOVA and multiple Tukey comparison tests and post-hoc analyses. The comparison before and after the same group was performed by paired sample *t* test. The count data were expressed as the number of cases (percentage) [n (%)], and the chi-square test was performed in parallel. If the distribution does not conform to the normal distribution, the Mann-Whitney U test was used. Changes in 3D gait quantitative parameters pelvic-lumbar imaging parameters and rehabilitation were calculated as delta (Δ)-parameter, and their correlation were tested using the Pearson correlation coefficient, and the test level was two-sided α = 0.05. For all statistical analyses in this study, the statistically significant difference was set at *P* = 0.05.

## Results

A prospective self-controlled study before-after surgery was performed on 85 patients with lumbar degenerative diseases. Kolmogorov-Smirnov normality test was applied for normality distribution test of variables. According to the results of normality test, kinematics, joint mobility and gait spatiotemporal parameters had normal distribution and analyzed by parametric tests, while the other variables were analyzed by non-parametric tests.

### Changes of gait time-space parameters in patients before and 6 months after operation

A statistically significant difference (*P* < 0.05) was seen between the step length, step speed, and step frequency six months after the operation and those before the operation. The difference was found to be higher than the previous values. The single support phase and step width after 6 months of operation were not significantly different from those before operation (*P* > 0.05) (Table 1).

**Table 1:** Changes of gait spatiotemporal parameters $\bar x$ in 85 patients before operation and 6 months after operation (± s)

time	Number	Stride duration (s)	Standing time(s)	Swing time(s)	Single support phase(%)	Dual support phase (%)	Stride length(m)	step size (m)	Pace (m/s)	Cadence (step/min)	Step width (m)
Before surgery	85	1.22 ± 0.13	0.75 ± 0.12	0.47 ± 0.05	38.70 ± 2.25	11.15 ± 3.48	0.99 ± 0.19	0.51 ± 0.15	0.82 ± 0.18	99.18 ± 9.27	0.10 ± 0.07
6-month post-surgery	85	1.09 ± 0.07	0.6 7 ± 0.13	0.42 ± 0.05	38.75 ± 3.02	10.21 ± 1.35	1. 22 ± 0. 1 9	0.6 1 ± 0.27	1.17 ± 0.13	105.83 ± 6.24 _	0.09 ± 0.03
*t* -value		8.118	4.169	6.519	0.122	2.322	7.892	2.985	14.433	5.490	0.784
*P* value		0.000	0.000	0.000	0.903	0.021	0.000	0.003	0.000	0.000	0.434

### Changes in kinematics-joint range of motion in 85 patients before and 6 months after surgery

The angles of pelvic tilt, pelvic rotation, hip adduction and abduction, and hip rotation were all found to be decreased post-surgery. Conversely, the angles of hip flexion, knee flexion, and knee extension were observed to be increased after the operation, with statistical significance (*P* < 0.05) (Refer to Table 2 for details).

**Table 2:** Changes in kinematics and joint mobility of 85 patients before surgery and 6 months after surgery ($\bar x$± *s*, °)

time	Number of cases	pelvic tilt	pelvic rotation	hip adduction and abduction	hip flexion	hip rotation	knee flexion
Before surgery	85	3.32 ± 2.57	6.37 ± 7.25	4.81 ± 3.02	12.59 ± 8.25	12.35 ± 9.15	10.62 ± 5.52
6 months after surgery	85	1.5 2 ± 1.35 _	4.24 ± 3.25	2.39 ± 1. 1 5	20. 19 ± 2.86 _	7. 95 ± 4.02 _	3 5 0.56 ± 5.16
*t* -value		5.717	2.472	6.904	8.025	4.059	30.430
*P* value		0.000	0.014	0.000	0.000	0.000	0.000

### Changes in pelvic-lumbar spine imaging parameters in patients before and 6 months after the operation

All patients underwent standing full-length lateral spine and lateral lumbar spine X-ray radiographs preoperatively and 6-month post-operatively in a standardized position to evaluate the preoperative and postoperative sagittal alignment parameters and Roussouly classification. The LL (44.1), DH (13.53), and ADH (17.56) of patients 6 months after operation were higher than those before operation, and the difference was statistically significant (*P* < 0.05). After 6 months, the PT (16.80), SS (31.71), PI (47.10), and PDH (9.06) of the patients were not significantly different from those before the operation (*P* > 0.05) (Table 3).

**Table 3:** Changes of pelvic-lumbar imaging parameters in patients before operation and 6 months after operation

time	Number of cases	PT (°)	LL (°)	SS (°)	PI (°)	DH (mm)	ADH (mm)	PDH (mm)
Before surgery	85	16.80 ± 4.73	34.74 ± 3.63	32.42 ± 4.56	46.86 ± 3.33	10.82 ± 0.97	13.31 ± 1.51	8.91 ± 1.82
6 months after surgery	85	16.80 ± 3.93	44.10 ± 5.74	31.71 ± 4.26	47.10 ± 3.24	13.53 ± 1.55	17.56 ± 3.01	9.06 ± 1.58
*t* value		0.010	12.706	1.051	0.497	13.686	11.660	0.545
*P* value		0.992	0.000	0.295	0.620	0.000	0.000	0.587

Abbreviations: pelvic tilt (PT); lumbar lordosis (LL); sacral slope (SS); pelvic incidence (PI); intervertebral space height (DH); anterior height of the intervertebral space (ADH); posterior height of the intervertebral space (PDH).

### Changes of VAS, JOA and ODI scores, the cross-sectional area of multifidus muscle in patients before and 6 months after operation

The muscle cross-sectional area was higher than that before the operation, and the difference was statistically significant (*P* < 0.05) (Table 4). The VAS score (2.43) and JOA score (23.09) of patients 6 months after operation were higher than those before operation (5.96 and 14.39, respectively), and the difference was statistically significant (*P* < 0.05). After 6 months, the ODI value (18.56) of the patients was significantly lower than those before the operation (29.85) (*P* < 0.05) (Table 3).

**Table 4:** Changes of VAS, JOA and ODI scores, multifidus cross-sectional area before operation and 6 months after operation in patients

time	Number of cases	VAS score	JOA score	ODI value	Multifidus cross-sectional area (mm^2^)
Before surgery	85	5.96 ± 1.43	14.39 ± 2.86	29.85 ± 3.72	267.85 ± 45.74
6 months after surgery	85	2.43 ± 1.03	23.09 ± 2.83	18.56 ± 3.33	343.13 ± 41.02
*t* -value		18.570	19.952	20.824	11.296
*P* value		0.000	0.000	0.000	0.000

Abbreviations: visual analog pain scale (VAS); Japanese Orthopedic Association (JOA); Oswestry Disability Index (ODI).

### Correlation analysis of 3D gait quantification parameters, pelvic-lumbar spine imaging parameters, and rehabilitation assessment scale

Pearson correlation analysis showed that VAS score, ODI value and stride duration, standing duration, swing duration, double support phase, pelvic Roll, pelvic rotation, hip adduction and abduction, and hip rotation angle were positively correlated (*r* > 0, *P* < 0.05), and negatively correlated with stride length, step length, pace speed, stride frequency, hip flexion, and knee flexion-extension angle (*r* < 0, *P* < 0.05); LL, DH, ADH, JOA score, multifidus muscle cross-sectional area and stride duration, stance duration, swing duration, dual stance, pelvic roll, pelvic rotation, hip adduction and hip rotation angles were negatively correlated (*r* < 0, *P* < 0.05), and positively correlated with stride length, step length, pace, stride frequency, hip flexion and knee flexion and extension angles (*r* > 0, *P* < 0.05) (Table 5).

**Table 5:** Correlation analysis of 3D gait quantitative parameters, pelvic-lumbar imaging parameters and rehabilitation assessment scale

Project	ΔLL	Δ DH	ΔADH	ΔVAS score	ΔJOA score	ΔODI value	Δ multifidus cross-sectional area
Δ stride duration	-0.374 ^**^	-0.424 ^**^	-0.368 ^**^	0.387 ^**^	-0.391 ^**^	0.467 ^**^	-0.279 ^**^
Δ Standing time	-0.205 ^**^	-0.255 ^**^	-0.198 ^**^	0.178 ^*^	-0.173 ^*^	0.235 ^**^	-0.191 ^**^
Δ Swing time	-0.351 ^**^	-0.446 ^**^	-0.390 ^**^	0.498 ^**^	-0.480 ^**^	0.497 ^**^	-0.293 ^**^
Δ double support phase	-0.485 ^**^	-0.512 ^***^	-0.482 ^**^	0.572 ^***^	-0.498 ^**^	0.621 ^**^	-0.384 ^**^
Δ stride length	0.414 ^**^	0.515 ^***^	0.401 ^**^	-0.478 ^**^	0.444 ^**^	-0.504 ^***^	0.444 ^**^
Δ step size	0.226 ^**^	0.343 ^**^	0.250 ^**^	-0.277 ^**^	0.298 ^**^	-0.326 ^**^	0.175 ^*^
Δpace _	0.512 ^***^	0.612 ^***^	0.526 ^***^	-0.528 ^***^	0.524 ^***^	-0.615 ^***^	0.521 ^***^
Δpace _	0.541 ^***^	0.635 ^***^	0.574 ^***^	-0.632 ^***^	0.416 ^**^	-0.685 ^***^	0.715 ^***^
ΔPelvic tilt	-0.725 ^***^	-0.652 ^***^	-0.485 ^**^	0.745 ^***^	-0.596 ^***^	0.741 ^***^	-0.485 ^**^
Δ pelvic rotation	-0.685 ^***^	-0.585 ^***^	-0.652 ^***^	0.615 ^***^	-0.635 ^***^	0.699 ^***^	-0.512 ^***^
ΔHip adduction and abduction	-0.578 ^***^	-0.635 ^***^	-0.584 ^***^	0.625 ^***^	-0.645 ^***^	0.711 ^***^	-0.536 ^***^
Δ hip flexion	0. 525 ^***^	0.516 ^***^	0.532 ^***^	-0.698 ^***^	0.486 ^**^	-0.698 ^***^	0.516 ^***^
Δ hip rotation	− 0.658 ^***^	-0.585 ^***^	-0.698 ^***^	0.635 ^***^	-0.615 ^***^	0.674 ^***^	-0.528 ^***^
ΔKnee flexion and extension	0.642 ^***^	0.638 ^***^	0.628 ^***^	-0.775 ^***^	0.810 ^***^	-0.785 ^***^	0.553 ^***^

Abbreviations: delta-lumbar lordosis (ΔLL); delta-intervertebral space height (Δ DH); delta-anterior height of the intervertebral space (ΔADH); sacral slope (SS); pelvic incidence (PI); posterior height of the intervertebral space (PDH); delta-visual analog pain scale (ΔVAS) score; delta-Japanese Orthopedic Association (ΔJOA) score; delta-Oswestry Disability Index (ΔODI) value. Note: delta (Δ) represents the difference before operation and 6 months after operation, ^*^P <0.05, ^**^P <0.01, ^***^P <0.001

## Discussion

In the present prospective self-controlled before-after study conducted on patients with lumbar degenerative disease (*n* = 85) admitted to our Hospital between September 2018 and December 2021, six months following surgery, patients’ LL, DH, and ADH were greater than prior to surgery levels, and the difference was statistically significant. The patients’ PT, SS, PI, and PDH at six months did not alter substantially from their before the operation values. The difference in the muscle cross-sectional area before and after the procedure was statistically significant. Prior to surgery, there was a decrease in the angles of the pelvic tilt, pelvic rotation, hip adduction and abduction, and hip rotation. Before the operation, the hip flexion, knee flexion, and extension angles were all higher, and the change was statistically significant.

In order to obtain the kinematics and electromyography signals of the human lower limbs and to research the function of human gait movement by analyzing its temporal and spatial properties, a range of sensor technologies are used in the field of gait analysis. Numerous gait characteristics, such as spatiotemporal parameters, dynamic joint range of motion, neuromuscular activity, and dynamic joint reaction force, can be obtained by gait analysis. To analyze the properties of a particular anatomical plane, these parameters can be assessed in two-dimensional (2-D) or three-dimensional (3-D) planes. Gait analysis is currently a highly recognized clinical tool that has been utilized and studied extensively in the treatment of hemiplegia, cerebral palsy, osteoarthritis in the knee, etc.

Nonetheless, adequate study in the real world on its use in spinal degenerative illnesses is currently lacking. Ultimately, our findings bolster the notion that 3-D gait quantitative analysis is a useful tool for assessing the effectiveness of surgery and the impact of rehabilitation training on patients suffering from lumbar degenerative illnesses. Additionally, it can serve as a resource for efficient patient evaluation training and aid in the future formulation of more precise and customized rehabilitation plans.

Spine and lumbar injuries are increasing due to aging, organ dysfunction, osteoporosis, and slow metabolism. External factors like work fatigue, physical labor, and traffic accidents also increase the risk. Without timely treatment, these conditions can reduce quality of life and disability rates. 3D gait analysis systems simplify operations and improve accuracy, aiding in the diagnosis, treatment, and evaluation of spine-related diseases. This study compared 3D gait quantitative parameters and conventional rehabilitation evaluation methods in 85 patients with lumbar degenerative diseases.

### Minimally invasive transforaminal interbody fusion improves gait spatiotemporal and kinematic parameters in patients with lumbar degenerative diseases

Zhong et al. assessed the ratio of left and right support as the observation index of walking balance and found that percutaneous transforaminal endoscopy combined with postoperative rehabilitation treatment can increase the ratio of postoperative support and improve the balance and symmetry of walking [59]. Later studies used a 3D gait analysis system to analyze the causes of walking impairment and indications for postoperative rehabilitation training in patients with lumbar spinal stenosis after lumbar percutaneous transforaminal surgery [59–62]. The results showed that rehabilitation training for pelvic anteversion could improve the patient’s gait index. Moreover, the stance ratio of the patient’s symptomatic lower limb increased by > 75% after surgery. may be some indications for targeted rehabilitation training after surgery. However, there is currently a lack of data support on the changes in gait spatiotemporal parameters and kinematic parameters of patients before and after minimally invasive transforaminal interbody fusion surgery. The results of this study found that the striding time, standing time, swinging time and double support phase of patients after surgery were lower than those before surgery, and the stride length, step length, pace speed, and stride frequency were higher than those before surgery, indicating that minimally invasive trans-vertebral The effectiveness of transforaminal interbody fusion combined with rehabilitation training can increase patients’ stride length, step length, pace speed, and step frequency, and improve walking balance and symmetry. Haddas et al. compared the back step length and gait speed of patients with degenerative scoliosis before and after surgery, and the results showed that both indexes increased significantly after surgery [63]. Miura et al. reported in a patient with low back pain and gait disorder caused by a congenital flat back syndrome that the patient’s gait speed, step length, and gait frequency were improved after operation [44] which were similar to the results of this study. Moreover, they reported that the gait scores on both sides of the patients after surgery were lower than those before surgery, and the gait deviation index was higher than before surgery, but there was still a certain gap compared with healthy people, suggesting that patients with lumbar degenerative diseases received surgery. The lumbar spine is in a state of self-protection for a short period of time and is difficult to fully return to normal levels. In this study, the pelvic tilt, pelvic rotation, hip internal and external, and hip rotation and retraction angles on both sides of the patients after surgery were all lower than those before surgery, and the hip flexion and knee flexion-extension angles were all increased, indicating that minimally invasive transforaminal interbody fusion Combined with postoperative phased rehabilitation training, it can increase the range of motion of the hip and knee joints, and improve the tilt and rotation of the pelvis. Accelerate the recovery of patients’ waist and abdominal functions and improve their balance ability when walking. Among them, bed rest and passive activities are mainly used in the first week after the operation, and active activities in bed are implemented in the second week, which can promote blood circulation in the lower extremities and avoid pressure sores and muscle atrophy. Active spinal stretching and gradual stretching of the lower back muscles 3–6 weeks after surgery can increase muscle flexibility and relieve pain and other clinical symptoms. After 6 weeks after the operation, intensive training and physical force training can improve the hip joint, knee joint and ankle joint, and improve the flexibility of the spine and the symmetry of the pelvis, thereby improving the walking ability. In addition, strengthening the hamstrings, gluteus maximus, thigh muscles and other muscle groups can not only improve the strength of the core muscles, but also improve the control and stability of the hip and knee joints.

Humans walk in various altered gaits when they are in pain, but the resulting mechanism has not been fully elucidated. It is mostly believed to be related to the theory of pain adaptation, that is, the muscle activity around the pain site will change during movement or isometric contraction. When the antagonist is used, the motor neuron output increases, which can reduce the maximum voluntary muscle contraction and speed, and the range of movement; when the muscle acts as an agonist, the motor neuron output decreases at this time. The purpose of the above-mentioned pain adaptation is to protect the injured part, and the motor control system can develop abnormal movement patterns through the action of long-term pain adaptation strategies. Watanabe et al. found that surgery combined with aerobic exercise for 3 weeks after surgery can reduce pain in patients with knee osteoarthritis and improve walking speed, stride length, and walking distance [64]. They concluded that the mechanism of gait improvement might be related to pain. Few studies have evaluated the fear-avoidance mental state and gait analysis of patients with lumbar disc herniation and reported that gait was moderately correlated with the fear-avoidance belief questionnaire [61, 65–67]. Symptoms such as pain appear after the stimulation, so there is a fear-escape psychology for walking, thereby prolonging the limb support phase and slowing down the pace. After the operation, the pain symptoms of the patient are relieved, and the patient’s support is improved, so the pace of the walk is accelerated after the operation. The step length is extended. Therefore, we believe that there are many mechanisms for the improvement of gait after pain relief, which still need to be further explored in clinical or basic experiments.

### Correlation between 3D gait quantification parameters and pelvic-lumbar imaging parameters

Studies have found that spine-pelvic dislocation balance and a series of parameter changes play an important role in the occurrence, development and outcome of lumbar degenerative diseases, and are helpful for further evaluation, diagnosis and treatment of diseases. When lumbar degenerative diseases occur, the pelvis undergoes corresponding anatomical changes in order to compensate for biomechanical changes. Roussouly et al. reported that patients with degenerative spondylolisthesis, compared with healthy people, had higher PI and SS values, often accompanied by a compensatory increase in LL values [68]. Barrey et al. reported that patients with degenerative lumbar disc herniation had lower SS values, TK values, and LL values, which indicated that the lumbar lordosis was small, the spine was tilted forward, and the spine was in an unbalanced state [69]. Zhang et al. compared the changes in spinal and pelvic imaging parameters in patients (*n* = 30) with degenerative lumbar spondylolisthesis before and after surgery [70]. Their findings showed that the LL value increased after surgery, and the degree of improvement of LL value before and after surgery was closely related to VAS score and ODI index. Ma et al. found that patients (*n* = 61) with lumbar degenerative diseases had significant changes in pelvic-lumbar imaging parameters before and after surgery [13]. It is not difficult to see from the above domestic and foreign studies that the imaging parameters of the pelvis and lumbar spine are closely related to the condition of lumbar degenerative diseases. In this study, the LL, DH, and ADH of patients after surgery were higher than those before surgery, similar to the results of the above study, further confirming that changes in pelvic-lumbar sagittal plane balance parameters can reflect the occurrence and development of diseases to a certain extent.

There are few reports at home and abroad on the relationship between 3D gait quantitative parameters and pelvic-lumbar sagittal plane balance parameters, and the correlation between them has not yet been clarified. In this study, further Pearson correlation analysis was used to find that LL, DH, and ADH were negatively correlated with striding time, standing time, swing time, dual support phase, pelvic tilt, pelvic rotation, hip adduction and abduction, and hip rotation angle (*r* < 0, *P* < 0.05), and it was positively correlated with stride length, step length, gait speed, stride frequency, hip flexion and knee flexion-extension angle (*r* > 0, *P* < 0.05), confirming that the 3D gait in patients with lumbar degenerative diseases There is a significant correlation between the quantitative parameters of posture and the sagittal balance parameters of the pelvis-lumbar spine. It is speculated that the reason may be that the change of gait after surgery may cause changes in the parameters of the spine-pelvis, which may cause the hip joint, knee joint, ankle joint, etc. of the lower extremities. Changes in parameters; during walking, uncompensated sagittal imbalance increases the anterior lever arm on the sacroiliac joint, causing the sacrum to rotate anteriorly; since the sacroiliac joint is a double joint, compensatory twisting motion (sacral Anterior rotation) is restricted, and uncompensated severe sagittal imbalance in turn leads to anterior pelvic tilt during walking by increasing the anterior lever arm at the sacroiliac joint.

### Correlation between 3D gait quantification parameters and conventional rehabilitation assessment methods

The VAS score, ODI index, JOA score, and multifidus cross-sectional area are the field indicators for evaluating the postoperative rehabilitation effects in patients with lumbar degenerative diseases. Among them, the ODI index has been used in clinical practice for more than 10 years, and some countries use this marker. The table serves as the gold standard for assessing the outcome of spinal surgery. In this study, the VAS score and ODI value of patients after surgery were lower than those before surgery, and the JOA score and multifidus cross-sectional area were higher than those before surgery, indicating that minimally invasive transforaminal interbody fusion combined with staged rehabilitation training is helpful. Reduce the pain level of patients with lumbar degenerative diseases; relieve low back pain and limb dysfunction. Pearson correlation results showed that VAS score, ODI value, JOA score, and multifidus cross-sectional area were significantly correlated with gait spatiotemporal parameters, and kinematics-joint range of motion, which confirmed the 3D gait quantification of patients with lumbar degenerative diseases. The parameters are closely related to the conventional rehabilitation evaluation methods, which further demonstrates that the parameters of the 3D gait quantitative analysis can be used in the postoperative rehabilitation evaluation of patients with spinal degenerative diseases, and have high application value. The possible reason is that the postoperative nerve compression in patients with lumbar degenerative diseases is relieved, the nerve function is gradually restored, and the gait is relieved. There is no obvious pain and numbness in the lower limbs after the operation, so the postoperative gait parameters, ODI, and VAS scores are all improved. Al- Obaidi et al. analyzed the correlation between gait characteristics and pain in 31 patients with chronic low back pain [71]. Their findings showed that the patient’s step length, walking speed, and single-support phase were significantly correlated with the degree of pain. Further stepwise regression analysis demonstrated that the expected pain is a risk factor for predicting deficits in fast walking speed, similar to the results of this study.

## Conclusion

This study explored how minimally invasive transforaminal intervertebral fusion can enhance lower limb motor function in patients with lumbar degenerative diseases post-surgery. It can increase stride length, pace, and stride frequency, as well as hip flexion, knee flexion, and extension angles. Additionally, 3D gait quantitative parameters in these patients are linked to pelvic-lumbar imaging parameters, VAS scores, JOA scores, and ODI values, making them useful for evaluating postoperative rehabilitation. A well-known phenomenon that rises with age is the fatty degeneration of multifidus muscle. Fat infiltration into muscles characterizes it [72, 73]. Considering the age, evaluation of the effect of fatty degeneration grades on rehabilitation parameters with a larger sample size is recommended for future studies.

## Data Availability

The data that support the findings of this study are available from the corresponding author upon reasonable request.
